# Combined effect of DBM, PRP, and bone marrow fluid on bone union in a rat posterolateral fusion model

**DOI:** 10.1038/s41598-023-41844-5

**Published:** 2023-09-12

**Authors:** Takuma Otagiri, Yasuhiro Shiga, Takashi Hozumi, Yusuke Matsuura, Ikuko Tajiri, Naoya Takayama, Maria Alejandra, Yuki Shiko, Sumihisa Orita, Kazuhide Inage, Yawara Eguchi, Takane Suzuki, Miyako Suzuki-Narita, Michiaki Mukai, Tomohito Mukaihata, Ryuto Tsuchiya, Soichiro Tokeshi, Kohei Okuyama, Takahito Arai, Noriyasu Toshi, Takeo Furuya, Satoshi Maki, Yasuchika Aoki, Seiji Ohtori

**Affiliations:** 1 Department of Orthopaedic Surgery, Graduate School of Medicine, Chiba University, Chiba, Japan; 2 Department of Regenerative Medicine, Graduate School of Medicine, Chiba University, Chiba, Japan; 3 Clinical Research Center, Chiba University Hospital, Chiba, Japan; 4 Chiba University Center for Frontier Medical Engineering, Chiba, Japan; 5 Department of Bioenvironmental Medicine, Graduate School of Medicine, Chiba University, Chiba, Japan; 6 Department of Orthopaedic Surgery, Eastern Chiba Medical Center, Chiba, Japan

**Keywords:** Stem cells, Medical research

## Abstract

Platelet-rich plasma (PRP) promotes bone union through osteoinduction. We investigated whether adding demineralized bone matrix (DBM), derived naturally from biomaterial and with various growth factors, for osteoconductivity and bone marrow fluid for osteogenesis results in different bone unions. Eight-week-old male Sprague–Dawley rats were divided into four groups of five based on transplantation material: sham control (C group); DBM alone (D group); DBM + PRP (DP group); and DBM + PRP + bone marrow fluid (DPB group). After posterolateral fusion at L3-5, postoperative weekly CT imaging determined average number of bone union in facet joints (4 joints × 5 animals = 20 joints) and bone formation. Pathological evaluation and bone strength were assessed using 3-point bending two weeks postoperatively. Facet joint bone union at four weeks postoperatively was 4/20 (20%, DP group) and 8/20 (40%, DPB group) joints. Six weeks postoperatively, it was 7/20 (35%, D group), 12/20 (60%, DP group), and 16/20 (80%, DPB group). Eight weeks postoperatively, it was 13/20 (65%, D group), 17/20 (85%, DP group), and 20/20 (100%, DPB group), suggesting that DPB > DP > D > C. Bone formation and bone strength showed a similar DPB > DP > D > C group trend. Adding PRP and bone marrow fluid to DBM promotes bone union and strength.

## Introduction

Lumbar spine arthrodesis is indicated for degenerative spinal conditions with instability, such as lumbar spondylolisthesis^1^. Combining spinal fusion with autogenous bone grafting, such as iliac or fibula grafting, allows for earlier bone union. Union at the site of bone grafting is a significant factor leading to excellent outcomes of spinal fusion. However, nonunion often causes complications such as pain, neuropathy, and implant failure^2^. In recent years, the development of implants has led to minimally invasive surgery and strong fixation; however, nonunion has still been reported. Furthermore, the use of autogenous bone is associated with problems such as pain at the donor site, fractures, and infection^3,4^. Hence, we need to develop methods that avoid these problems and achieve good spinal fusion results.

Artificial and allogeneic bone grafts are often used as alternatives to autogenous bone grafts in spinal surgery. Demineralized Bone Matrix (DBM), a type of artificial bone used in Europe and the United States for a long time, became available in Japan in April 2019. DBM uses demineralized human bone cortex as grafted bone, and high bone union rates have been reported^5,6^. In addition to its "osteoconductive capacity" as a scaffold for bone union, it has been reported to contain growth factors, such as transforming growth factor-beta (TGF-β), vascular endothelial growth factor (VEGF), and bone-forming proteins, such as BMP2 in trace amounts^7^. In addition, reports indicate that bone marrow fluid possesses osteogenic and osteoinductive properties that promote bone union^8^. Bone marrow fluid is often collected from the autologous iliac bone, and unlike the autologous iliac bone, there is no fracture risk. Platelet-rich plasma (PRP), which has abundant osteoinductive properties, is increasingly used in various fields. In PRP, whole blood is centrifuged and divided into platelet-poor plasma (PPP), PRP, and red blood cells; however, only the PRP layer is collected. Like bone marrow fluid, PRP contains various growth factors, such as platelet-derived growth factor (PDGF), TGF-β, and VEGF, which are believed to promote the differentiation of osteoblasts, preosteoblasts, and other cells. Numerous animal and clinical studies have demonstrated its effectiveness in stimulating bone union^9,10^.

The successful achievement of bone union requires the presence of osteoconductivity, osteoinduction, and osteogenesis. We hypothesized that the combination of DBM for osteoconductivity, bone marrow fluid for osteogenesis, and PRP for osteoinduction would promote bone union. It is important to note that while bone marrow fluid contains growth factors conducive to bone induction, previous research by Schmidmaier et al. demonstrated that the concentration of these growth factors in PRP is significantly higher than that in bone marrow fluid^11^. This highlights the enhanced potential of PRP in facilitating the differentiation of osteoblasts and other bone-forming cells. These material advantages also include avoiding the risk of infection and fractures^12,13^. However, there have been only a few reports on the effects of these combinations on bone union. This novel study analyzed the impact of DBM, PRP, and bone marrow fluid on bone union.

## Materials and methods

### Experimental animals

Male Sprague–Dawley rats were used in this study, and their body weights were between 250 and 300 g. The study protocols for animal procedures follow the National Institutes of Health Guidelines for the Care and Use of Laboratory Animals (2011 revision). The ethics committee of Chiba University approved the study protocol, and this study is reported in accordance with ARRIVE guidelines (PLoS Bio 8(6), e1000412,2010).

### PRP preparation

This study used allograft blood instead of autograft blood^14^. Eight-week-old male Sprague–Dawley rats were injected intraperitoneally with anesthetics (medetomidine hydrochloride [0.06 mg], midazolam [0.8 mg], butorphanol tartrate [1.0 mg], and saline [0.58 ml]) to produce sedation and analgesia. After anesthesia, approximately 15 mL of fresh blood was collected transcardially with a syringe containing 2 mL of acid-citrate-dextrose solution A (Terumo, Tokyo, Japan) to prevent clotting. The collected blood was centrifuged (KN70; Kubota, Tokyo, Japan) at 1500 rpm for 10 min, separating the plasma fraction from erythrocytes, which was further centrifuged at 3000 rpm for 10 min to pellet platelets, as per previous investigations^15^. PRP necessitates activation before its use by introducing a calcium chloride solution (1 mEq/mL; Otsuka Pharmaceutical, Tokyo, Japan) and a thrombin solution (Mochida Pharmaceutical, Tokyo, Japan), each of which was one-tenth of the PRP amount. The platelet counts in fresh PRP and whole blood was determined using a hematology analyzer.

### Bone marrow fluid assessment

**Figure 1:**
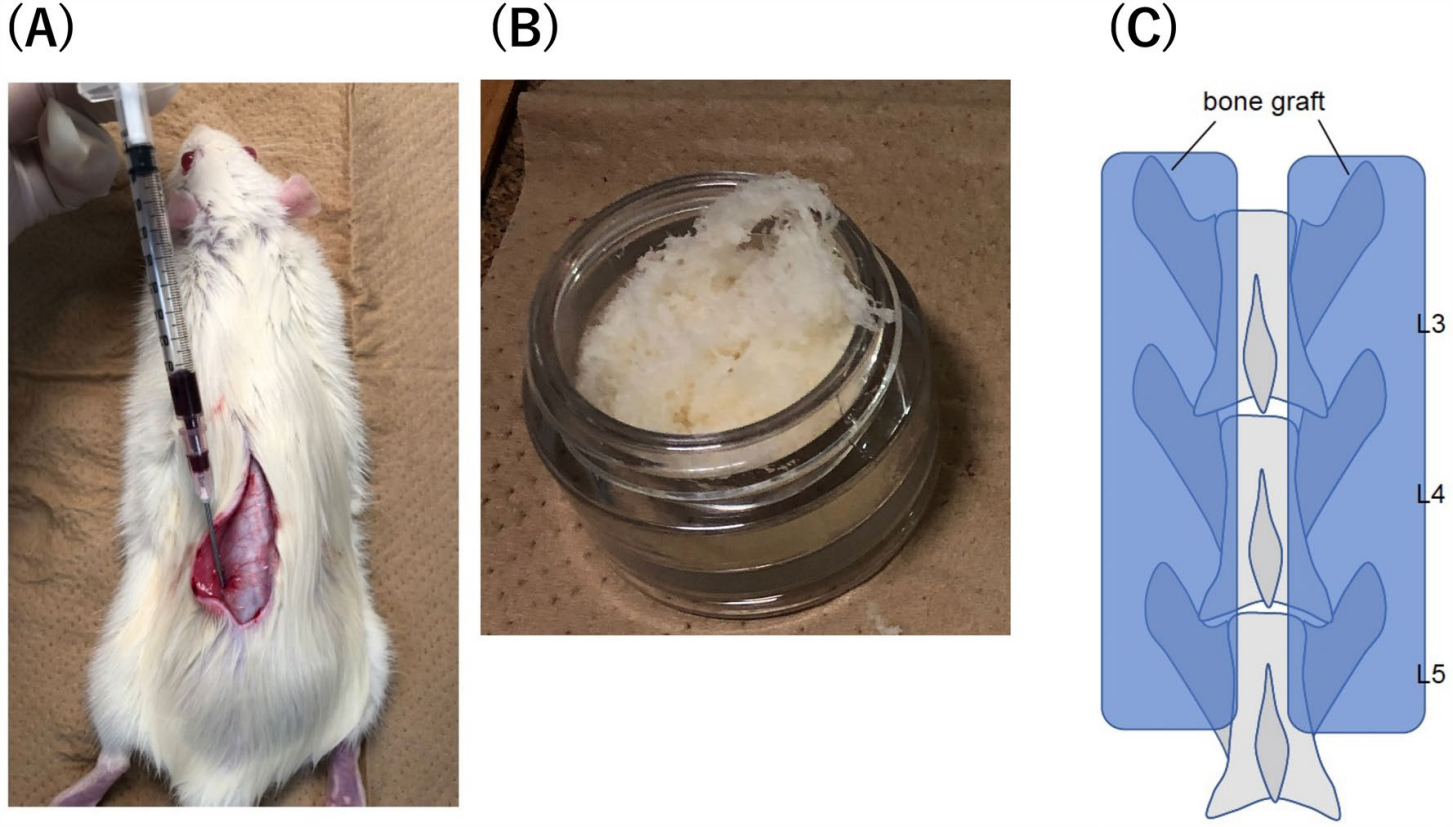
(**A**) Bone marrow fluid collection. Approach the iliac bone through the same skin incision as the bone graft and collect with an 18G needle and syringe. (**B**) Demineralized bone matrix. Fibrous structure; 0.5 ml was used for each rat. (**C**) The 3rd/4th/5th posterolateral lumbar fusion (PLF) surgery.

Bone marrow fluid was obtained through autologous transplantation. The iliac bone was exposed during surgery, and an 18 G needle was used for puncturing to aid bone marrow fluid extraction. May-Giemsa staining was performed on all five blood samples to confirm the presence of bone marrow fluid (Fig. 1a). First, samples were smeared, dried, fixed with methanol, and allowed to dry naturally. Second, the cells were stained with Giemsa staining solution (Muto Corporation, Tokyo, Japan) for 1 h, washed, dried, and observed under a fluorescence microscope (BZ-X800, Keyence Corp.). Moreover, to characterize the hematopoietic progenitor population, three blood samples were harvested from bone marrow and peripheral blood and treated with ammonium chloride for 20 min to lyse the red blood cells. Then, the cells were centrifuged and stained with CD34-PE (Abcam, cat:ab223930). After 30 min of incubation on ice, the cells were resuspended with PBS + 2% FBS + 0.1% Propidium Iodide. Then, the cells were analyzed with BD FACS CantoII (BD Bioscience).

### Lumbar posterolateral fusion model

Spinal surgery was performed on 20 eight-week-old male Sprague–Dawley rats^10,13^. The 20 rats were divided into groups of five based on the graft material used: control group (C group), artificial bone group (D group), artificial bone with PRP group (DP group), aritificial bone with PRP, and bone marrow fluid group (DPB group). Group C was only exposed at L3-5 and did not undergo bone grafting. A DBM, fibrous Grafton® (Medtronic, Tokyo, Japan), was used as the artificial bone graft substitute (Fig. 1b). The DP group received a mixture of Grafton (0.5 mL) and gel-activated PRP (0.5 mL gel-activated PRP). In the DPB group, 0.5 mL of Grafton, 0.5 mL of gel-activated PRP, and 0.2 ml of bone marrow fluid were mixed and transplanted. No corticotomy was performed in any of the groups. Each rat’s bilateral posterolateral lumbar spine was exposed through a midline skin incision followed by two paramedian fascial incisions using blunt dissection to expose the bilateral lamina and transverse processes of L3–L5 (Fig. 1c). The transplantation of graft materials to the Sprague–Dawley rats was performed by a skilled spine surgeon. Equal amounts of graft material were transplanted on both sides of the L3-5.

### Radiographic examination (evaluation of bone union)

**Figure 2:**
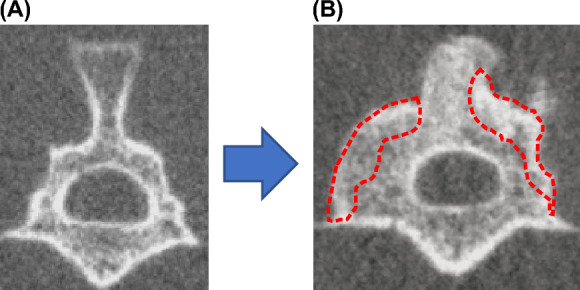
(**A**) Measurement of the volume of bone formation using Image J. (**B**) Determination of bone fusion by CT imaging.

Computed tomography (CT) (in vivo micro-CT system, R_mCT2, Rigaku Co.) imaging was performed under inhalation anesthesia with isoflurane (1.5% isoflurane; Mylan) at 2, 4, 6, and 8 weeks after surgery. A CT image of a coronal section through the center of the L4 transverse process of the lumbar vertebra was used to measure and record the new bone formation area compared with the preoperative image (Fig. 2). ImageJ software (NIH) was used to measure bone formation. Bone union of the facet joints at L3-5 and the bone graft area were also evaluated. Bone union was defined as the cross-linking of the facet joints^16^. A total of four bilateral L3-5 facet joints were counted for the number of joints in which bony fusion was obtained, and the average number of bone union in facet joints in each group was then calculated. Two independent observers who were blinded to the experiment determined the bone union, and the union was accepted if all three observers agreed.

### Histological examination

Following euthanasia, the lumbar vertebrae were obtained, and a paraffin block was created using 10% neutral buffer formalin (0.1 M, pH 7.4) for fixation. Transverse sections, which were 2-μm-thick, were then produced and stained with hematoxylin and eosin^14^. Images of the transected posterolateral fusion (PLF) region were created using brightfield microscopy (BZ-X800, Keyence Corp.).

### Mechanical strength examination (three-point bending test)

**Figure 3:**
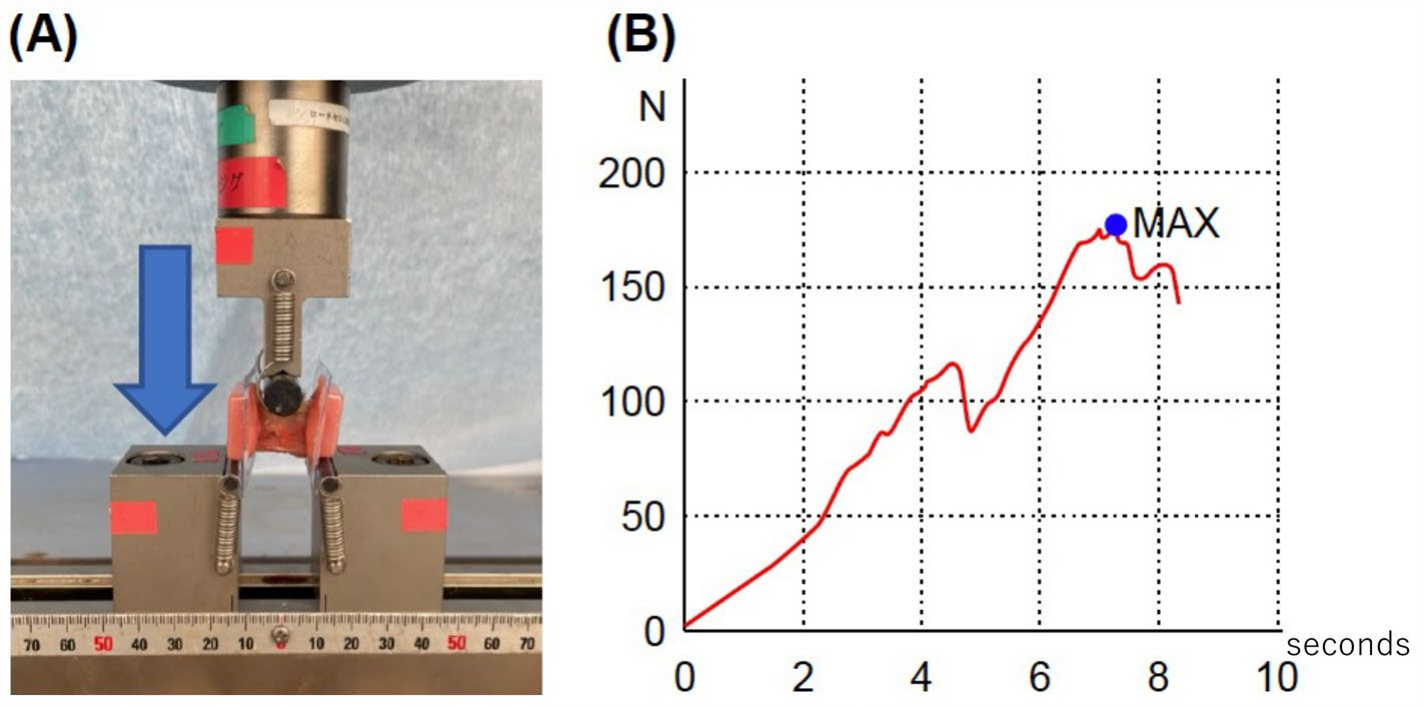
Mechanical strength examination; three-point bending test. (**A**) Three-point bending. (**B**) Representative plotting for initial peak pressure measurement.

L3–L5 lumbar spine specimens (3.5 cm long) were excised from rats two weeks after the operation. The samples were secured on both sides with plastic holders. A three-point bending test apparatus (Shimadzu, Tokyo, Japan) was used to conduct the tests. The compression strength of the dorsal side of the spine with PLF was determined by applying force to the ventral side at two points and the dorsal side at one point. To maintain the stiffness of the specimens, they were brought to room temperature on the day of euthanization. The samples were continuously monitored while pressure was applied during the experiments. There was no occurrence of rotation or slippage of the models. A preload of 10 N of force was applied to the bones at a rate of 0.1 mm/s, and a 10-s acclimation period followed^14^. The mechanical strength was defined as newtons at the point of rupture, as shown in Fig. 3.

### Statical analyses

Platelet contents of plasma and PRP were compared using Student’s *t*-test. Average number of bone union in facet joints, bone formation, and mechanical strength among the four groups (C, D, DP, and DPB) were tested using ANOVA and Dunnett’s and Tukey’s tests as post hoc tests. ﻿The criterion for significance was set at *p* < 0.05.

## Results

### Confirmation of PRP

The mean platelet count in the blood was 900 ± 124 × 10^3^/μL, and that in the PRP was 4028 ± 1228 × 10^3^/μL. On average, the platelet count in the PRP was approximately 4.47 times higher than that in the blood, which is a significantly higher concentration (*p* < 0.05).

### Growth factor concentrations

The mean concentration of PDGF-BB was 10.7 ± 2.5 ng/mL in the PRP. The mean concentration of TGF-β 1 was 618.5 ± 132.9 ng/mL in the PRP (Table 1).

**Table 1:** Concentrations of growth factors.

Growth Factor	Concentration (ng/ml)
PDGF-BB	10.7 ± 2.5
TGF-β1	618.5 ± 132.9

PDGF-BB, platelet-derived growth factor-BB; TGF-β1, transforming growth factor-β1.

### Bone marrow fluid

**Figure 4:**
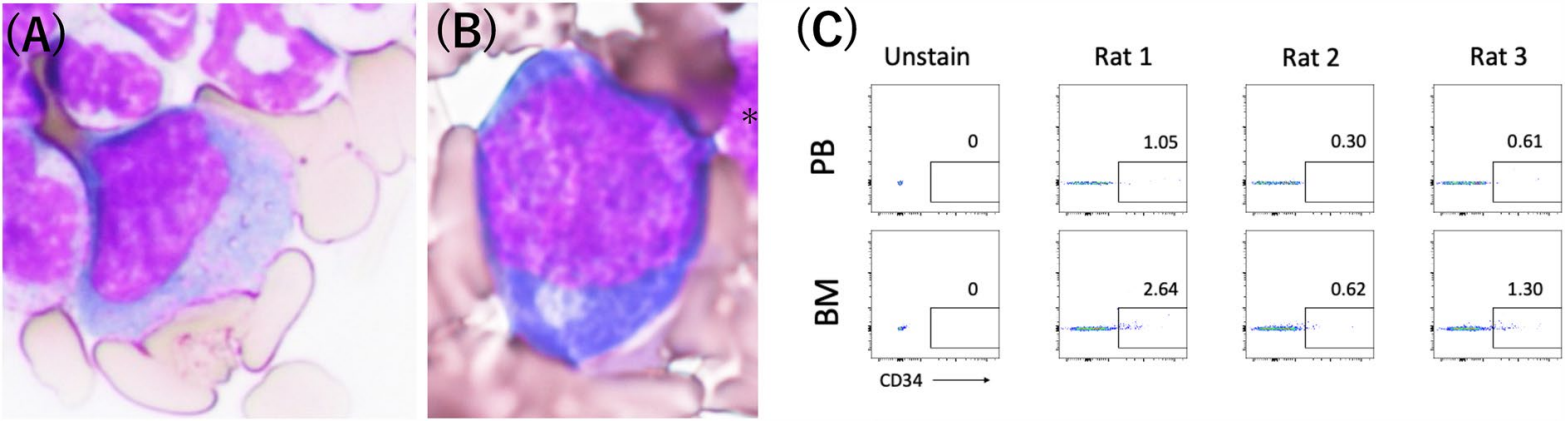
Evaluation of bone marrow fluid. (**A**) Promyelocyte with May-Giemsa staining. (**B**) Erythroblast with May-Giemsa staining. (**C**) FACS plot of bone marrow and peripheral blood.

May-Giemsa staining was performed on the bone marrow fluid rich in immature cells, including promyelocytes and erythroblasts (Fig. 4a,b). CD34^+^ cells were found in 0.3–1.05% of peripheral blood and 0.62–2.64% of bone marrow fluid. In all rats, the percentage of CD34^+^ cells in bone marrow fluid was more than twice that in peripheral blood (Fig. 4c). These results suggest that bone marrow fluid may be reliably collected and added to the transplanted bone.

### Average number of bone union in facet joints

**Figure 5:**
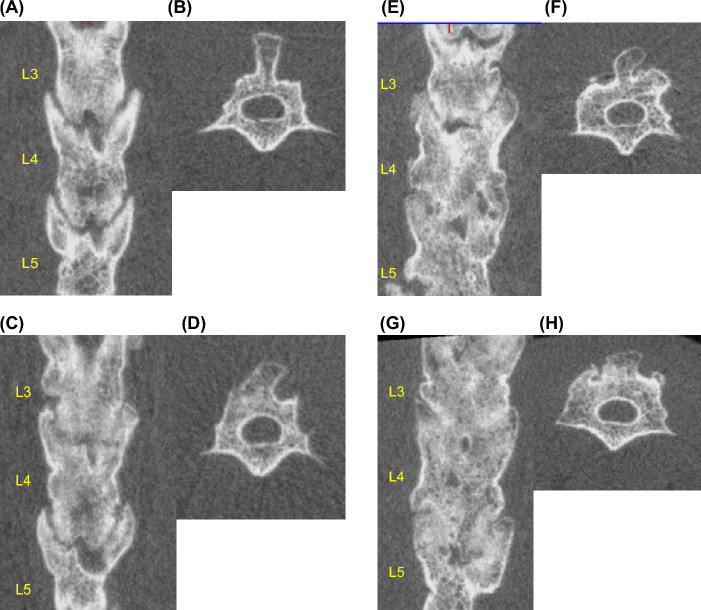
Representative CT sections at week 8 postsurgery. (**A**) Coronal section, C group. (**B**) Axial section of the 4th lumbar vertebral arch, C group. (**C**) Coronal section of the bone union, D group. (**D**) Axial section of the 4th lumbar vertebral arch, D group. (**E**) Coronal section of the bone union, DP group. (**F**) Axial section of the 4th lumbar vertebral arch, DP group. (**G**) Coronal section of the bone union, DPB group. (**H**) Axial section of the 4th lumbar vertebral arch, DPB group.

**Figure 6:**
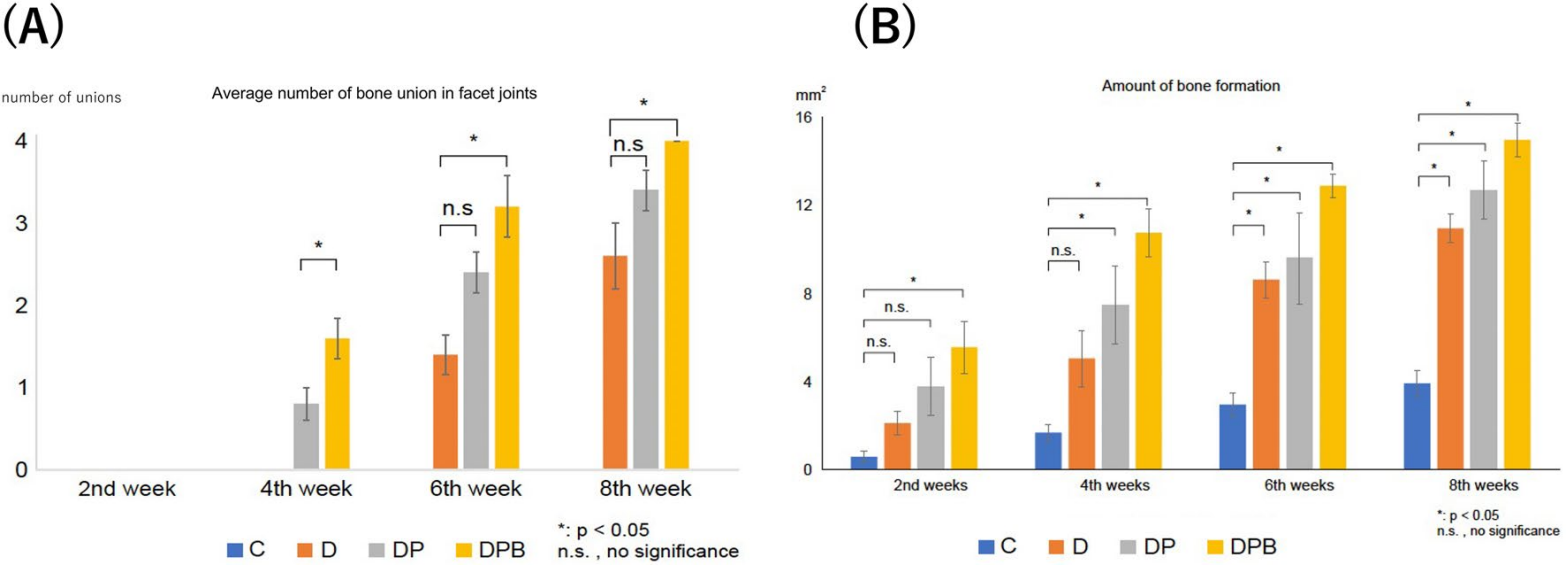
(**A**) Bone union rate between the facet joints in the four groups after surgery. (**B**) Amount of bone formation eight weeks after the surgery.

Primary CT images for each group are presented in Fig. 5. At two weeks, no facet joint fusion was observed in any group. At four weeks, there was no bone fusion in groups C and D; however, 4/20 joints (20%) and 8/20 joints (40%) in the DP and DPB groups, respectively, showed bone fusion, which was a significant difference between the DP and DPB groups (*p* < 0.05). At six weeks, no bone fusion was observed in group C; however, 7/20 joints (35%) in group D, 12/20 joints (60%) in group DP, and 16/20 joints (80%) in group DPB showed bone fusion, and there was a significant difference between the D and DPB groups (*p* < 0.05) but not between the D and DP groups (*p* = 0.06) or between the DP and DPB groups (*p* = 0.16). At eight weeks, group C had no bone fusion; group D had bone fusion in 13/20 joints (65%), group DP had bone fusion in 17/20 joints (85%), and group DPB had bone fusion in 20/20 joints (100%), with a significant difference between groups D and DPB (*p* < 0.05). However, no significant differences were observed between groups D and DP (*p* = 0.11) or between groups DP and DPB (*p* = 0.30) (Fig. 6a).

### Amount of bone formation

The area of new bone formation measured using ImageJ software was 0.56 ± 0.54 mm^2^ in group C, 2.10 ± 1.18 mm^2^ in group D, 3.76 ± 2.98 mm^2^ in group DP, and 5.58 ± 2.65 mm^2^ in group DPB at two weeks, showing a significant difference between groups C and DPB (*p* < 0.05) At four weeks, the values were 1.66 ± 0.86 mm^2^ in group C, 5.05 ± 2.85 mm^2^ in group D, 7.45 ± 3.95 mm^2^ in group DP, and 10.75 ± 2.45 mm^2^ in group DPB, showing a significant difference between groups C, DP, and DPB. At six weeks, the values were 2.94 ± 1.21 mm^2^ in the C group, 8.61 ± 1.85 mm^2^ in the D group, 9.58 ± 4.60 mm^2^ in the DP group, and 12.89 ± 1.16 mm^2^ in the DPB group, showing significant differences between the C, D, DP, and DPB groups (*p* < 0.05). At eight weeks, the area was 3.91 ± 1.27 mm^2^ in the C group, 10.95 ± 1.47 mm^2^ in the D group, 12.69 ± 2.49 mm^2^ in group DP, and 14.98 ± 1.69 mm^2^ in the DPB group, all of which showed significant differences compared with group C (*p* < 0.05), as at six weeks. At all points, bone formation was highest in the DPB group, followed by the DP, D, and C groups (Fig. 6b).

### Histological examination

**Figure 7:**
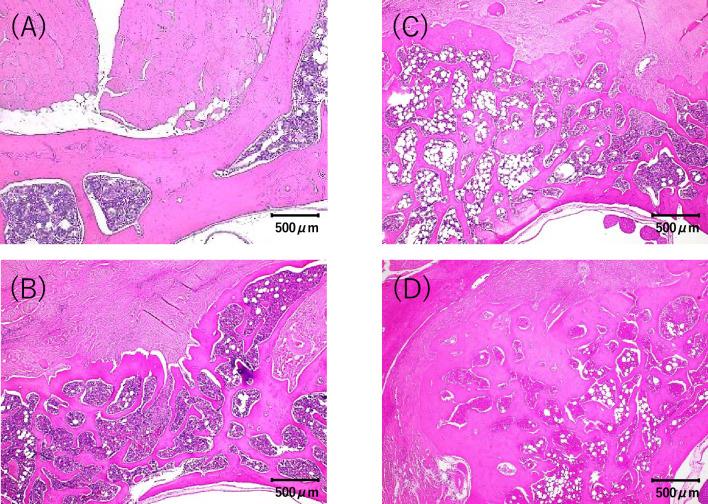
Histological images of the bone graft area. (**A**) Control group. (**B**) Artificial bone alone group. (**C**) Artificial bone with PRP group. (**D**) Artificial bone with PRP and bone marrow fluid group. Artificial bone promotes bone formation, and the addition of PRP and bone marrow fluid further increases bone formation.

In each group, the bone graft area was replaced with new bone tissue. The histological image in Fig. 7 shows the right vertebral arch of the fourth lumbar vertebra of a rat at 8 weeks postoperatively. While new bone formation was observed in all groups except group C, a trend toward bone formation was seen in the order of DPB group > DP group > D group, consistent with the area results measured by ImageJ.

### Mechanical strength examination (three-point bending test)

**Figure 8:**
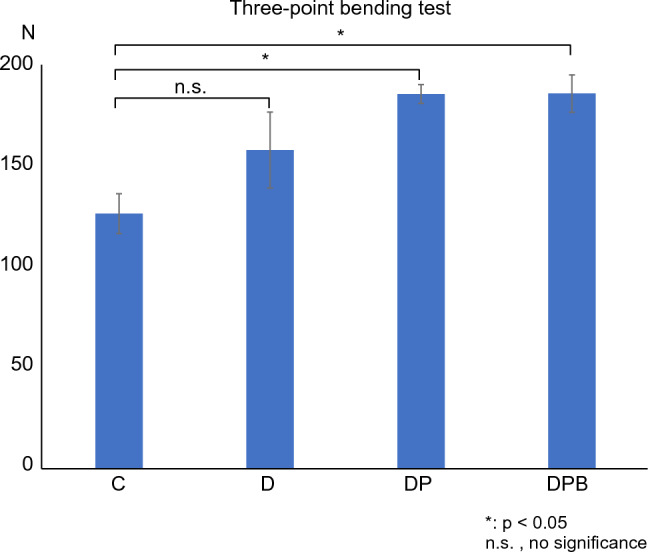
Mechanical strength evaluation using a three-point bending test. The addition of artificial bone, PRP, and bone marrow fluid will increase strength.

The results of the strength measurements are listed in Fig. 8. The mean pressure at the highest load was 126.2 ± 22.0 N for Group C, 157.75 ± 42.0 N for Group D, 185.51 ± 10.3 N for Group DP, and 185.75 ± 18.4 N for Group DPB. Although there was a significant difference between groups C, DP, and DPB (*p* < 0.05), there was no significant difference between the other groups. However, a trend toward greater intensity existed in groups C < D < DP < DPB.

## Discussion

A rat PLF model was created to evaluate the differences in bone union when PRP, bone marrow fluid, and DBM were combined. PRP and bone marrow fluid of assured quality were used. The results showed that combining DBM, PRP, and bone marrow fluid resulted in the highest average number of bone union in facet joints, bone formation, and bone strength. On the other hand, group D had an enhanced number of bone union in facet joints and bone strength compared with group C but was significantly lower than group DP.

Bone union requires three factors: osteoconductive ability as a scaffold for bone formation, osteogenic ability of osteoblasts, and osteoinductive ability to induce and differentiate into osteogenic cells^17^. Reports indicate that DBM functions as a promising scaffold (osteoconductive ability)^18,19^. Here, DBM promoted bone union compared with the control group. However, DBM alone results in poor bone formation and induction. Therefore, we hypothesized that combining DBM with PRP and bone marrow solution containing DBM may result in better quality and earlier bone union, as demonstrated in this study.

To the best of our knowledge, the first study on PRP for bone union was published by Marx et al. in 1998^20^, and it stated that platelet counts should be concentrated to approximately 4–5 times that of whole blood for PRP to promote bone union. Weibrich et al. reported that a platelet concentration of 2–6 times that of whole blood is required for PRP to have a bone union effect in vivo^20^. In this study, the average platelet concentration of PRP was 4.47 times that of whole blood, making it an optimum concentration.

Numerous studies have been published on PRP bone fusion to the spine, showing its effectiveness not only at the experimental animal level but also in clinical studies^14,22,23^. Kubota et al. reported a 22% increase in the bone union rate and a reduction of approximately two months in the duration of bone union when 62 patients with lumbar spine disease were divided into 31 PRP-treated and 31 PRP non-treated groups and treated with PLF^9^. Conversely, there are reports of reduced union rates of autologous bone with platelet gel concentrate added and controversies regarding PRP bone union^24,25^. However, many studies did not analyze platelet counts or growth factor concentrations in PRP, which may not meet the recommended dosage.

There are literature reports of earlier bone union^10,14^. However, they are based on radiographic evaluation, which may make it difficult to distinguish between osteogenesis and bone fusion. We believe that CT imaging at each observation point and assessment by the two examiners allowed for a more accurate determination of bone fusion.

In this study, May-Giemsa staining and measurement of CD34^+^ cells in bone marrow fluid were performed to ensure that bone marrow fluid was collected. A previous report indicated that CD34 cells are found in the bone marrow, where they represent 1% to 4% of mononuclear cells^26^. In our study, a similar percentage of CD34-positive cells was found, suggesting that optimal collection was achieved. Both PRP and bone marrow fluid contain growth factors that can promote bone union. However, as highlighted by Schmidmaier’s findings, the concentration of these growth factors in PRP is greater than that in bone marrow fluid^11^. While bone marrow fluid alone can promote bone union, the inclusion of PRP, with its rich concentration of growth factors, is believed to enhance this process. Our study’s findings, where the DPB group demonstrated earlier bone union compared to the DP group, support this hypothesis.

Our results suggest that the combination of DBM, PRP, and bone marrow fluid led to increased bone formation and earlier bone union. We hypothesize that the early-stage cross-linking of the facet joints by bone resulted in more osteogenic activity, thereby leading to increased fracture resistance.

While several research studies^9,10,14^ have demonstrated that DBM, PRP, and bone marrow fluid can accelerate bone union, no previous studies have combined these three elements. Furthermore, this study evaluated the pathology and bone strength, demonstrating the superiority of the combined use of these two techniques from multiple perspectives. In conclusion, this study suggests that adding PRP and bone marrow fluid to DBM results in more effective bone union and strength.

### Limitations

This study has several limitations. One limitation is that pathological tests were not quantified. Although quantifying bone formation and strength demonstrated the effectiveness of adding PRP and bone marrow fluid, it was insufficient for pathological evaluation. Therefore, further investigation is required in the future. Another limitation concerns PRP quality. Although PDGF-BB and TGF-β 1 were calculated in this study, other growth factors were not examined. Since various growth factors are involved in bone healing, it will also be necessary to investigate other growth factors.

Furthermore, the datasets used and analysed during the current study are available from the corresponding author upon reasonable request.

## References

[1] Chan AK, Sharma V, Robinson LC, Mummaneni PV (2019). Summary of guidelines for the treatment of lumbar spondylolisthesis. Neurosurg. Clin. N. Am..

[2] Tsutsumimoto T, Shimogata M, Yoshimura Y, Misawa H (2008). Union versus nonunion after posterolateral lumbar fusion: A comparison of long-term surgical outcomes in patients with degenerative lumbar spondylolisthesis. Eur. Spine J..

[3] Kim DH (2009). Prospective study of iliac crest bone graft harvest site pain and morbidity. Spine J..

[4] Dimitriou R, Mataliotakis GI, Angoules AG, Kanakaris NK, Giannoudis PV (2011). Complications following autologous bone graft harvesting from the iliac crest and using the RIA: A systematic review. Injury.

[5] Brecevich AT (2017). Efficacy comparison of Accell Evo3 and Grafton demineralized bone matrix putties against autologous bone in a rat posterolateral spine fusion model. Spine J..

[6] Aghdasi B, Montgomery SR, Daubs MD, Wang JC (2013). A review of demineralized bone matrices for spinal fusion: The evidence for efficacy. Surgeon.

[7] Berven S, Tay BK, Kleinstueck FS, Bradford DS (2001). Clinical applications of bone graft substitutes in spine surgery: Consideration of mineralized and demineralized preparations and growth factor supplementation. Eur. Spine J..

[8] Johnson RG (2014). Bone marrow concentrate with allograft equivalent to autograft in lumbar fusions. Spine.

[9] Kubota G (2019). Platelet-rich plasma enhances bone union in posterolateral lumbar fusion: A prospective randomized controlled trial. Spine J..

[10] Kamoda H (2013). The effect of platelet-rich plasma on autologous. J Bone Joint Surg Am..

[11] Schmidmaier G (2006). Quantitative assessment of growth factors in reaming aspirate, iliac crest, and platelet preparation. Bone.

[12] Noh T (2021). Bone marrow aspirate in spine surgery: case series and review of the literature. Cureus.

[13] Lechner R, Putzer D, Liebensteiner M, Bach C, Thaler M (2017). Fusion rate and clinical outcome in anterior lumbar interbody fusion with beta-tricalcium phosphate and bone marrow aspirate as a bone graft substitute. A prospective clinical study in fifty patients. Int. Orthop..

[14] Shiga Y (2016). Freeze-dried platelet-rich plasma accelerates bone union with adequate rigidity in posterolateral lumbar fusion surgery model in rats. Sci. Rep..

[15] Crovetti G (2004). Platelet gel for healing cutaneous chronic wounds. Transfus. Apher. Sci..

[16] Kim G (2022). Bone union-promoting effect of romosozumab in a rat posterolateral lumbar fusion model. J. Orthop. Res..

[17] Albrektsson T, Johansson C (2001). Oste**,** oinduction, osteoconduction and osseointegration. Eur. Spine J..

[18] Betz RR, Lavelle WF, Samdani AF (2010). Bone grafting options in children. Spine.

[19] Buser Z (2016). Synthetic bone graft versus autograft or allograft for spinal fusion: A systematic review. J. Neurosurg. Spine..

[20] Marx RE (1998). Platelet-rich plasma: Growth factor enhancement for bone grafts. Oral Surg. Oral Med Oral Pathol. Oral Radiol. Endod..

[21] Weibrich G, Hansen T, Kleis W, Buch R, Hitzler WE (2004). Effect of platelet concentration in platelet-rich plasma on peri-implant bone regeneration. Bone.

[22] Imagama S (2017). Efficacy of early fusion with local bone graft and platelet-rich plasma in lumbar spinal fusion surgery followed over 10 years. Glob. Spine J..

[23] Manini DR, Shega FD, Guo C, Wang Y (2020). Role of platelet-rich plasma in spinal fusion surgery: systematic review and meta-analysis. Adv. Orthop..

[24] Carreon LY, Glassman SD, Anekstein Y, Puno RM (2005). Platelet gel (AGF) fails to increase fusion rates in instrumented posterolateral fusions. Spine.

[25] Boden SD (2003). Efficacy of autologous growth factors in lumbar intertranverse fusions: Point of view. Spine.

[26] Siena S (1989). Circulation of CD34+ hematopoietic stem cells in the peripheral blood of high-dose cyclophosphamide-treated patients: Enhancement by intravenous recombinant human granulocyte-macrophage colony-stimulating factor. Blood.

